# The self online: When meaning-making is outsourced to the cyber audience

**DOI:** 10.1371/journal.pone.0294990

**Published:** 2023-12-20

**Authors:** Qi Wang, Angel Khuu, Miryam Jivotovski

**Affiliations:** Department of Human Development, Cornell University, Ithaca, New York, United States of America; The Hong Kong Polytechnic University, HONG KONG

## Abstract

This study examines the cyber audience’s perception of social media users’ persona based on their online posts from a cognitive meaning-making perspective. Participants (N = 158) answered questions about their personal characteristics and provided their 20 most recent Facebook status updates. Two groups of viewers, who viewed either the text-only or multimedia version of the status updates, answered questions about the Facebook users’ personal characteristics. The viewers’ perceptions of Facebook users deviated from the users’ self-perceptions, although user characteristics that serve social motives were more accurately perceived. Multimedia viewers were more accurate than text viewers, whereas the latter showed a greater consensus. Gender and ethnic differences of Facebook users also emerged in online person perceptions, in line with gendered and cultured characteristics. These findings shed critical light on the dynamic interplay between social media users and the cyber audience in the co-construction of a digitally extended self.

## Introduction

Our sense of self and identity is externalized in the digitally mediated world. The proliferation of social media and the internet over the past decades has introduced new modes of self-representation and expression. The selfhood comprises not only self-representations in the private mind of the individual but also self-presentations on social media platforms public to the cyber audience [1]. These self-presentations, achieved through sharing personal experiences and opinions online in text and other media forms (e.g., photo, video, hyperlink), are frequently motivated by the need to connect with others and to express oneself [2–7]. They constitute a digital extension of the selfhood unique to the social media age. Little is known, however, about how social media users’ self-presentations through online posts are viewed by other social media users, or the cyber audience. Understanding online person perception is critical to facilitate effective communication and interpersonal relationships [8–10]. The present study set out to examine to what extent the personal characteristics of social media users in the eyes of the cyber audience via meaning making of online posts reflect the users’ views of themselves.

### Characteristics of self-presentation in online posts

Posting personal experiences and opinions on social media is a ubiquitous daily activity for many. With the unlimited audience and the permanent storage for the shared information in cyber space, this activity has become an important means for individuals to achieve social bonding and personal expression [4–7]. Accordingly, social media users often, intentionally or subconsciously, create online posts with content and form in service of their personal and social motives. Research has shown that social media users tend to be open and disclosive in their posts, revealing intimate personal details and innermost thoughts and feelings, and that online self-disclosure is associated with extraversion and disclosiveness traits [4, 11, 12]. Social media users also frequently use tactics such as directly asking questions or seeking feedback to encourage immediate interaction from the cyber audience, which creates a sense of connectedness with the audience [13, 14]. Furthermore, social media users often selectively post positive experiences online to glorify themselves and create an ideal self-image [15–17]. In addition, when writing posts, social media users tend to craft their stories to highlight captivating aspects of their experiences and portray a unique persona with maximized individuality and relational orientation [6, 18, 19].

Thus, to achieve self-expression and social connection, the self as presented through social media posts may be characterized by heightened extraversion, disclosiveness, sense of connectedness, self-esteem, independence, and interdependence [1, 6]. Indeed, posts with these characteristics have been shown to have positive consequences for the social media users’ social network and psychological well-being [1, 20, 21]. The lack of these characteristics, in contrast, tends to be associated with negative influences of social media use [15, 22, 23]. Furthermore, the functional capabilities of social media platforms provide rich technology features in multimedia forms beyond simple text, such as photo, animation, video, hyperlink, and even livestreaming and augmented reality. The multimedia features are expected to be effective means for individuals to present their personal characteristics in social media posts that best serve their goals to communicate the self and to relate to other selves online [6, 19].

Importantly, from a cognitive perspective, individuals form a sense of self and personality through a constructive process of meaning-making of their lived experiences [6, 24–26]. By interpreting, evaluating, and making sense of their day-to-day encounters (e.g., receiving an A+ in a difficult exam), individuals construct coherent stories of who they are, their values and beliefs, and their unique personal characteristics (i.e., I’m a smart, hard-working person who is aiming towards a successful career). This meaning-making process may take place in the private space—within the person—when one reflects on life events, or in the shared space—between persons—when one tells elaborate personal stories to others. During the self-presentation in online posts, however, the meaning-making process is no longer just located “within” or “between,” but externalized: It is outsourced to the cyber audience [1, 6, 27].

### The role of the cyber audience

**Fig 1:**
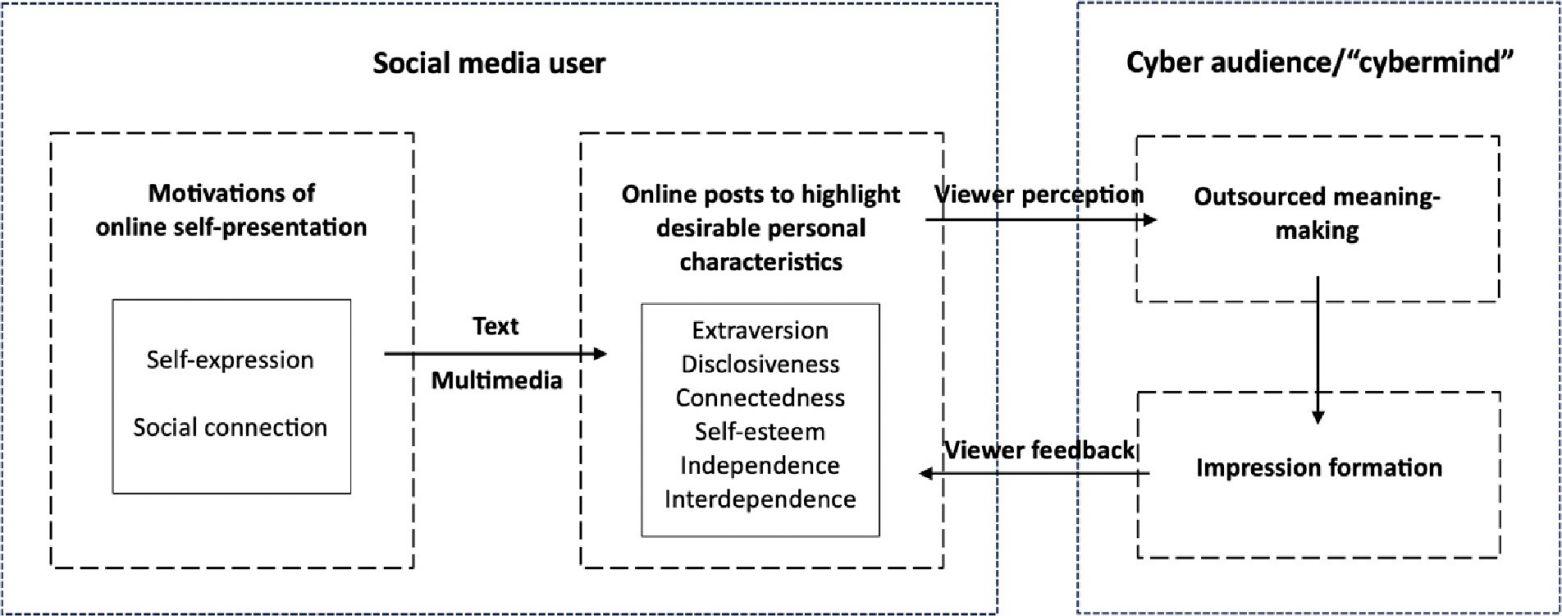
Self-presentation and outsourced meaning-making on social media.

Does the cyber audience perceive the personal characteristics of the social media users based on the users’ self-presentation in online posts? Prior research on personality perception based on personal websites (e.g., blogs) or online profiles has shown that observers generally have high levels of accuracy and consensus in their impressions of social media users [10, 28, 29]. However, unlike personal blogs or profiles in which social media users explicitly convey who they are, online posts such as Facebook status updates lack a coherent framework about the author. Other than being chronologically ordered and having the author as the main character, social media posts are typically isolated episodes and states of mind that the author experiences, with the earlier posts bearing no obvious logical connections with the later ones. Also, social media posts are often casually generated brief messages with little contemplation [30], where the author may hardly engage in meaning making about themselves. Thus, to work out who the author is, the cyber audience needs to make sense of the disparate experiences shared in the social media posts and interweave the slices of information into a coherent representation about the author [1, 6, 27]. In other words, the meaning-making process is outsourced to the cyber audience, who encodes, integrates, and updates information from social media posts about the characteristics, roles, and experiences of the author [1, 6]. This constructive meaning-making process may be further shared among the cyber audience members and function through the transactive mind of the virtual community, or the “cybermind” [31, 32]. Fig 1 illustrates our theoretical model.

Interestingly, social media posts with some of those characteristics described above (e.g., disclosiveness, self-esteem, sense of connectedness) tend to engage a larger and more responsive cyber audience who provides comments, feedback, and likes to the posts [22, 33, 34]. The responsiveness of the cyber audience, in turn, encourages more frequent and more open sharing of such posts on social media [2, 34, 35]. Thus, it appears that the cyber audience may be able to infer and “appreciate” the distinct characteristics–at least some of them—of the social media users via online posts. Nonetheless, it is unknown how accurate the cyber audience’s perceptions of the social media users’ personal characteristics based on the online posts are in comparison with the users’ self-views, what factors influence the perceptions, and how consistent the perceptions are among the members of the cyber audience. In addition, although multimedia tools can be utilized by social media users in online posts, it is unclear in the outsourced meaning-making context how they may differentially influence the accuracy and consensus of the cyber audience’s perceptions of the social media users’ persona.

### The present study

A main goal of the present study is to examine the personal characteristics of social media users as perceived by the cyber audience based on online posts, in comparison with the social media users’ views of themselves. Different from prior research that has focused on the perception of the Big Five traits from personal blogs and profiles [10, 28, 29], this study focuses on personal characteristics that are often presented via social media posts for social and personal purposes [1, 20, 21]. Participants provided self-descriptions and completed a battery of measures for their personal characteristics, including extraversion, disclosiveness, connectedness, self-esteem, independence, and interdependence. They then submitted the 20 most recent status updates from their Facebook accounts. Two groups of viewers (i.e., the cyber audience) rated the status updates of the participants (i.e., Facebook users) on the same dimensions of personal characteristics as in the participants’ self-reports. Given that the cyber audience needs to make sense of the Facebook users’ status updates to infer the users’ persona, we hypothesized that discrepancies would occur between the cyber audience’s perceptions of the Facebook users’ characteristics and the users’ self-views (H1). We further explore whether the discrepancies would vary by characteristics such that the cyber audience might overestimate characteristics that seem easier to infer from the content of the posts (e.g., disclosiveness) [34]; while underestimate those that may be less obvious from the posts (e.g., independence, interdependence) (H2).

A second goal of the study is to examine how social media posts in simple text and those in multimedia forms would differentially influence the cyber audience’s meaning making about the social media users’ persona. The two groups of viewers rated the same Facebook status updates that were either text-only or in multimedia forms (i.e., text plus other media forms). Based on research showing that different types of information online are helpful for observers to form accurate impressions [36, 37], we expected that status updates in multimedia forms would be more facilitative for the cyber audience to detect the Facebook users’ communicative intent and infer the characteristics of the users’ online self-presentations than would text-only status updates (H3). However, multimedia status updates, with rich information and user interface, might contribute to more diverse interpretations and views among the cyber audience members about the Facebook users’ persona, when compared with text-only status updates (H4). In other words, multimedia status updates would increase accuracy in viewer perceptions but undermine viewer consensus in the outsourced meaning-making process.

In addition, a third goal of the present study is to examine whether demographic factors, particularly gender and ethnicity, would influence how the cyber audience perceives the personal characteristics of social media users (H5). Gendered ideology and cultural beliefs shape how individuals view and present themselves [6, 38, 39], which may be reflected in their Facebook posts. Thus, in line with the cultured and gendered self-construal where women and Asians exhibit a greater relationship orientation than men and Westerners such as White Americans who exhibit a greater autonomous orientation [38, 40, 41], we expected that the cyber audience would perceive a greater sense of connectedness and interdependence in female and Asian Facebook users and greater independence in male and White Facebook users. In addition, given that women tend to score higher on extraversion than men [42] and Westerners tend to score higher on extraversion than Asians [43, 44] we expected the cyber audience to perceive greater extraversion in female and White than male and Asian Facebook users, respectively, The cyber audience would also perceive greater self-esteem in White than Asian Facebook users, given the stronger emphasis on positive self-views in Western than Asia cultures [45–47].

## Method

### Participants

A total of 158 undergraduate students (*Mean age* = 20.20 years) at Cornell University participated in the study, including 75 White Americans, 41 Asians, 11 African Americans, 20 Hispanics, and 11 multiracial participants based on self-reported information. Among the participants, 115 self-reported as female and 43 self-reported as male. Only current Facebook users were eligible to participate. Participants received extra course credits for their participation. Based on prior research on personality perception on social media [10, 29], a medium effect size was used to determine the sample size. A power analysis using G*Power [48] showed that a sample size of 92 participants would be needed to achieve a power of 0.95 to detect effects with a size of *f* = 0.20 and α = 0.05. Given that this was the first study to examine perceptions of personal characteristics via online posts, we recruited a larger sample to ensure sufficient power.

### Procedure and measures

**Fig 2:**
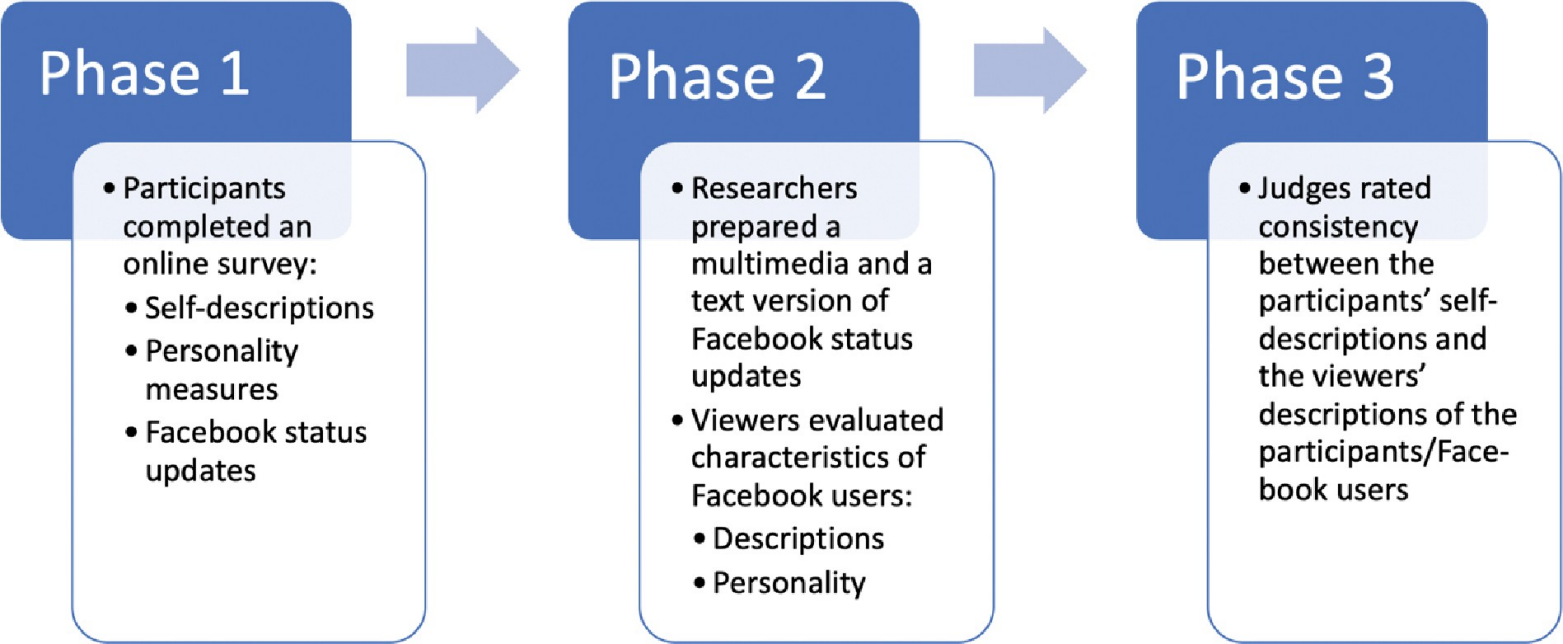
The study phases.

The data collection consisted of three phases, as illustrated in Fig 2.

#### Phase 1

Participants completed an online survey via Qualtrics. They were asked to describe who they were and complete a battery of scale measures for their personal characteristics, which are detailed below. Then participants were asked to submit their 20 most recent status updates from their Facebook accounts. They were instructed to take a screenshot of each of the 20 posts and submit them for later coding.

*Self-descriptions*. A shortened version of the Twenty Statement Test [49, 50] was used to elicit the participants’ descriptions about themselves. Participants were instructed to provide 10 unique responses to the prompt: “Who am I?” They were asked to provide the responses in the order as they came to mind and not to worry about logic or “importance.”

*Extraversion*. Extraversion was measured by 4 items adopted from prior research [12, 51], including “I like to have a lot of people around me,” “I really enjoy talking to people,” “I like to be where the action is,” and “I usually prefer to do things alone” (reverse coded). Participants rated each statement on a 7-point scale from 1 (*strongly disagree*) to 7 (*strongly agree*). Higher mean scores across the items indicated greater extraversion, with Cronbach’s alpha = .77 in the current sample.

*Disclosiveness*. The participants’ disclosive tendency was measured with a shortened version of Wheeless’ Revised Self-Disclosure Scale (RSDS) [52], which includes 5 items (e.g., “I often disclose intimate, personal things about myself without hesitation.”) measuring disclosiveness as a trait [4, 12]. Participants rated each item on a 7-point scale from 1 (*strongly disagree*) to 7 (*strongly agree*). Higher mean scores across the items indicated greater disclosiveness, with Cronbach’s alpha = .81 in the current sample.

*Connectedness*. A scale by Sheldon et al. (2011) [14] was used to measure the participants’ sense of connectedness with others, including 3 positive (connection) items (e.g., “I felt a sense of contact with people who care for me, and whom I care for”) and 3 negative (disconnection) items (e.g., “I felt unappreciated by one or more important people”). Participants rated their experience “during the last week” on a scale from 1 (not true) to 9 (very true). Negative items were reverse scored such that higher mean scores across the items represented greater relationship closeness. Cronbach’s alpha = .62 in the current sample.

*Self-esteem*. Self-esteem was measured by the Rosenberg Self-Esteem Scale (RSES) [53], which consists of 10 items (e.g., “I feel that I have a number of good qualities”). Participants rated each item on a scale from 1 (*strongly disagree*) to 4 (*strongly agree*). Negatively worded statements were reverse-scored so that higher mean scores across the items indicated higher self-esteem, with Cronbach’s alpha = .88 in the current sample.

*Independence and interdependence*. Participants’ endorsement of independent versus interdependent self-views were assessed using the Self-Construal Scale (SCS) [54]. The scale consists of 30 statements to tabulate independent/autonomous orientation (e.g., "I enjoy being unique and different from others in many respects") and interdependent/relational orientation (e.g., "I usually go along with what others want to do, even when I would rather do something different"). For each statement, participants indicated their agreement or disagreement on a scale from 1 (strongly disagree) to 7 (strongly agree). Each participant received two mean scores, one for the strength of independence and one for the strength of interdependence. Cronbach’s alpha = .69 for both subscales in the current sample.

#### Phase 2

**Fig 3:**
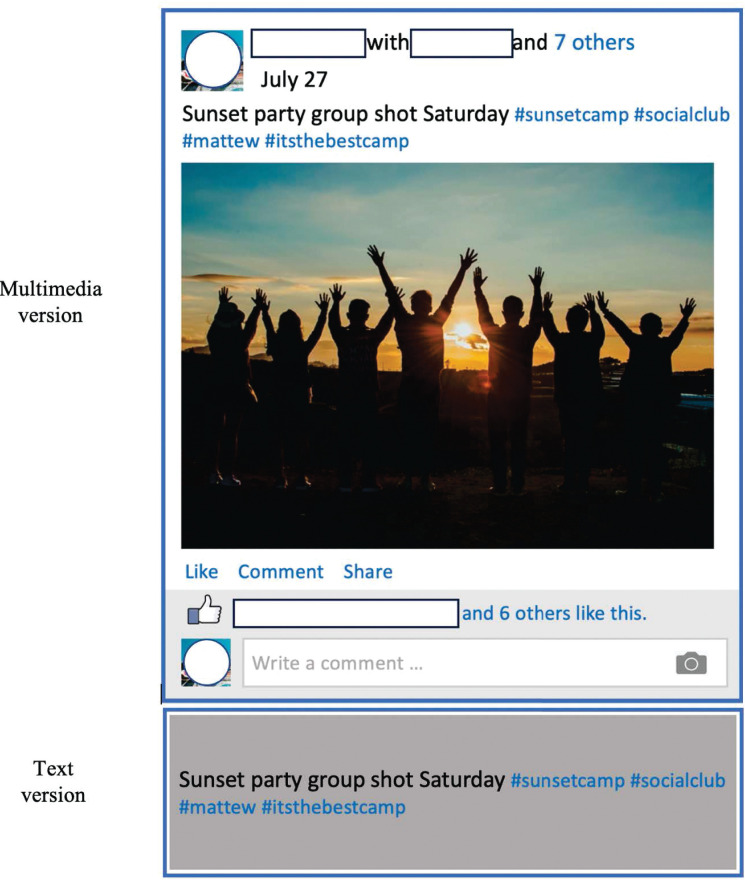
A simulated example of the multimedia and text versions of a Facebook status update in the study. Photos from https://www.pexels.com.

The participants’ Facebook status updates were first de-identified, with names and other identifiable information blocked. They were then prepared into two versions: a multimedia version as they were submitted, which could include text, pictures, links, and other media formats, and a text version where the text portion of the status updates was extracted (see Fig 3 for a simulated example). Then two groups of 6 “viewers,” who were Cornell University undergraduate students and who were blind to the study hypotheses, were randomly assigned to read either the multimedia or text version of the Facebook status updates. The viewers were research apprentices who received course credits for their work on the project. The number of viewers was determined by following prior research of online personality perception [10, 29]. Each viewer provided independent evaluation of each participant/Facebook user. First, the viewers were asked to describe who the Facebook user was, in a procedure paralleling the self-description task [49, 50]. The viewers were instructed to provide 10 unique responses to the prompt, “Who is he/she?” based on their reading of the participant’s Facebook status updates. They were asked to provide the responses in the order as they came to mind and not to worry about logic or “importance”.

After completing the descriptions, the viewers were asked to rate the Facebook user’s personal characteristics on 7-point scales, including extroversion (1 = very introvert, 7 = very extrovert); disclosiveness (1 = little, 7 = very disclosive); connectedness/relationship closeness (1 = not close, 7 = very close); self-esteem/positivity of self-views (1 = very negative, 7 = very positive); independence/self-centeredness (1 = very little, 7 = very much); and interdependence/relational orientation (1 = very little, 7 = very much).

#### Phase 3

Three “judges,” who were undergraduate research apprentices and who were blind to the study hypotheses, coded the consistency between the participants’ self-descriptions and the viewers’ descriptions of the participants based on their Facebook status updates. Instructions were provided to the judges following prior research [40, 55]. Any information elements (i.e., any nouns, verb phrases, or unique modifiers) that appeared in both self-descriptions and viewer descriptions were considered **consistent**. In general, elements were considered consistent if a participant and a viewer used the same or similar words to describe the same real-world entity (e.g., the participant responded, “I’m kind,” and the reviewer responded, “He is nice.”). Elements that were directly contradictory or that could not refer to the same real-world entity were considered **inconsistent** (e.g., the participant responded, “I’m kind,” and the viewer responded, “He is mean.”). In addition, elements that were not part of the participants’ self-descriptions but appeared in the viewers’ descriptions about the participants were coded as **commission,** whereas elements that were part of the participants’ self-descriptions but not mentioned in the viewers’ descriptions were coded as **omission**. The 3 judges independently coded a randomly selected set of data for inter-judge reliability estimates, which was good to excellent at Cronbach’s alpha = .90 for consistent, .76 for inconsistent, .90 for commission, and .90 for omission. Disagreements were resolved through discussion among the judges, who then split the data to complete the consistency coding.

### Ethics statement

Data and research materials of the study can be accessed at https://osf.io/h3anr/?view_only=a7f2871bbf0f48ca9b92a6f83530863a. This study was approved by the Cornell University Institutional Review Board (IRB) for Human Participants (Protocol ID#1206003146). Participants indicated their agreement to participate in a written consent at the initiation of the study.

## Results

In the following sections, we first compared ratings on personal characteristics made by participants themselves (i.e., Facebook users) and by the two groups of viewers (i.e., the cyber audience) (H1, H2, H3). Gender and ethnicity were considered in the analyses to examine whether these demographic factors would moderate the consistency between participant self-reports and viewer ratings (H5). We then examined the consistency between participants’ self-descriptions and the descriptions provided by the text versus multimedia viewers about the participants (H3). Finally, we examined viewer consensus, analyzing the consistency within each viewer group in how individual viewers perceived the participants’ persona based on meaning making of their Facebook status updates (H4). A few participants did not answer all the questions. The degree of freedom thus varied slightly across tests.

### Personal characteristics rated by Facebook users and the cyber audience

Each participant’s personal characteristics were rated 3 times: by self-report and by the two viewer groups based on the participant’s Facebook status updates (i.e., the multimedia group and the text group). Note that viewers made ratings on 7-point scales, so did participants who completed most measures of personal characteristics on 7-point scales except for connectedness and self-esteem. To make the ratings comparable, the latter two scales were converted to 7-point Likert scales (e.g., participants’ self-esteem scores were divided by 4 and then multiplied by 7). The means and standard deviations of the ratings by participants themselves and the two viewer groups are presented in Table 1.

**Table 1:** Means and standard deviations of personal characteristics.

	Self-report		Multimedia Viewer			Text Viewer		
	(N = 158)		(N = 151)			(N = 152)		
Characteristics	Mean	SD	Mean	SD	Corr.	Mean	SD	Corr.
Extroversion	4.90^a^	1.09	4.85^a^	0.83	0.28***	4.61^b^	0.85	0.23**
Disclosiveness	3.61^a^	1.22	4.42^b^	0.83	0.12	4.21^c^	1.12	0.09
Connectedness	4.95^ab^	0.96	5.02^a^	0.82	-0.02	4.77^b^	1.06	0.04
Self-esteem	5.48^a^	1.02	5.27^b^	0.47	0.11	4.91^c^	0.58	0.16*
Independence	4.89^a^	0.64	4.83^a^	0.67	-0.03	4.21^b^	0.73	-0.04
Interdependence	4.81^a^	0.61	4.15^b^	0.88	0.05	4.48^c^	1.04	0.16*

*Note*: Significant differences in mean ratings of each characteristic are denoted by different superscripts.

Corr. = Correlations between respective viewer ratings and self-reports.

p* < .05, p** < .01, p*** < .001

Correlations between self-reports and viewer ratings were also presented in Table 1. Overall, the consistency between individual Facebook users’ self-reported personal characteristics and the cyber audience’ judgments was low, with only the correlation for extroversion being significant for both viewer groups and the correlations for self-esteem and interdependence being significant for the text group.

To further examine the extent to which the cyber audience ascribes characteristics to Facebook users relative to users themselves, a repeated-measures ANOVA was conducted to examine the rater effect on each characteristic, comparing the ratings by participants themselves and by the two viewer groups. Then gender and ethnicity were separately entered into the model following each main analysis to examine whether gender or ethnicity moderated the consistency between participant self-reports and viewer ratings. The analyses involving ethnicity only included White and Asian participants given the small number of participants in other ethnic groups. Note that across all analyses, the main effect of rating groups remained the same pattern with or without gender or ethnicity in the model.

#### Extroversion

**Fig 4:**
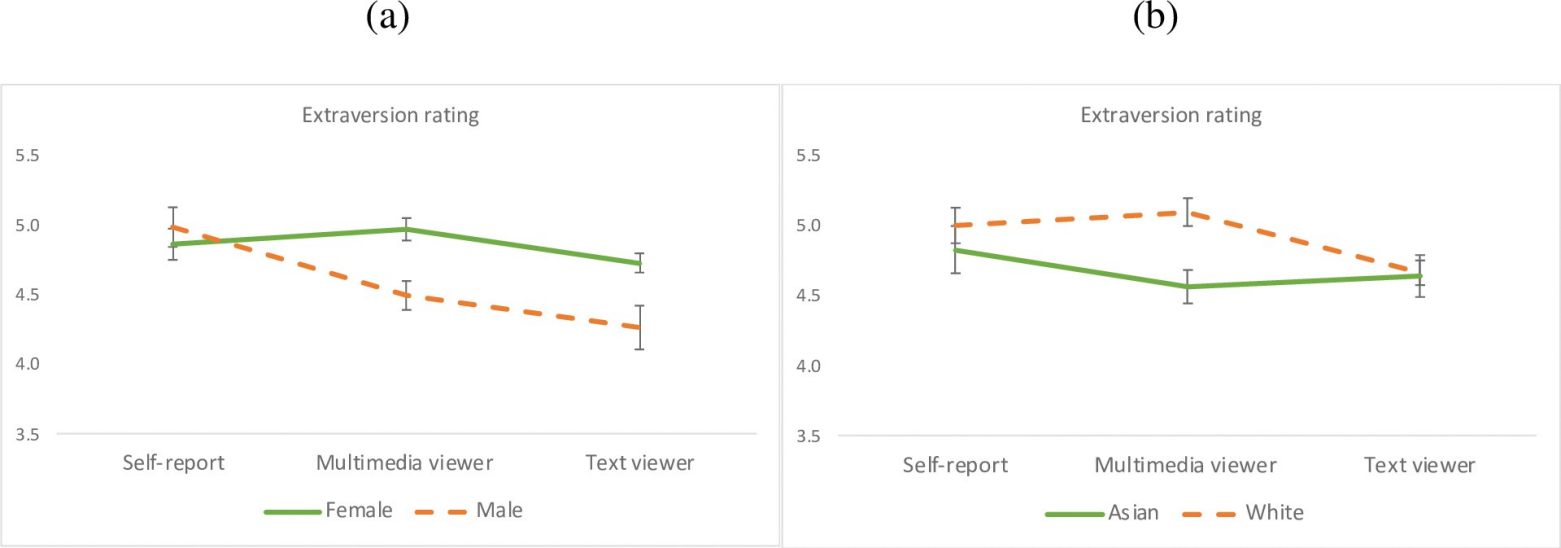
Interaction effects of rater group by (a) gender and (b) ethnicity on extraversion. Error bars represent the standard errors of the means. Both viewer groups, but not participants themselves, rated women higher than men on extroversion. The multimedia group, but not the text group or participants themselves, rated White Facebook users higher than Asians on extroversion.

A significant rater effect emerged for extroversion, *F*(2, 149) = 6.67, *p* = .0017, η_p_^2^ = .082, which was driven by the lower rating of the text group than the participants’ self-report, *F*(1, 151) = 7.49, *p* = .007, η_p_^2^ = .047, and the multimedia group, *F*(1, 150) = 10.97, *p* = .0012, η_p_^2^ = .068, who did not differ significantly from each other. When gender was entered into the model, a significant main effect of gender, *F*(1, 149) = 5.46, *p* = .021, η_p_^2^ = .035, and a Gender x Rater interaction emerged, *F*(2, 148) = 3.62, *p* = .029, η_p_^2^ = .047. Both viewer groups rated women higher on extroversion than men (*ps* < .01), who did not differ in their self-reports. When ethnicity was entered into the main model, a significant Ethnicity x Rater interaction emerged, *F*(2, 109) = 4.22, *p* = .017, η_p_^2^ = .072, whereby the multimedia group rated White participants higher on extroversion than Asians (*p* < .01), who did not significantly differ in their self-reports or in ratings by the text group. The interaction effects by gender and ethnicity are plotted in Fig 4.

#### Disclosiveness

A significant rater effect emerged for disclosiveness, *F*(2, 148) = 29.79, *p* < .0001, η_p_^2^ = .29, whereby both the multimedia and text groups provided higher ratings than did participants themselves, *F*(1, 149) = 52.80, *p* < .0001, η_p_^2^ = .26, *F*(1, 150) = 22.88, *p* < .0001, η_p_^2^ = .13. The multimedia group had higher ratings than the text group, *F*(1, 150) = 8.80, *p* = .0035, η_p_^2^ = .055. Gender and ethnicity had no significant main effect or interaction on disclosiveness ratings.

#### Connectedness

**Fig 5:**
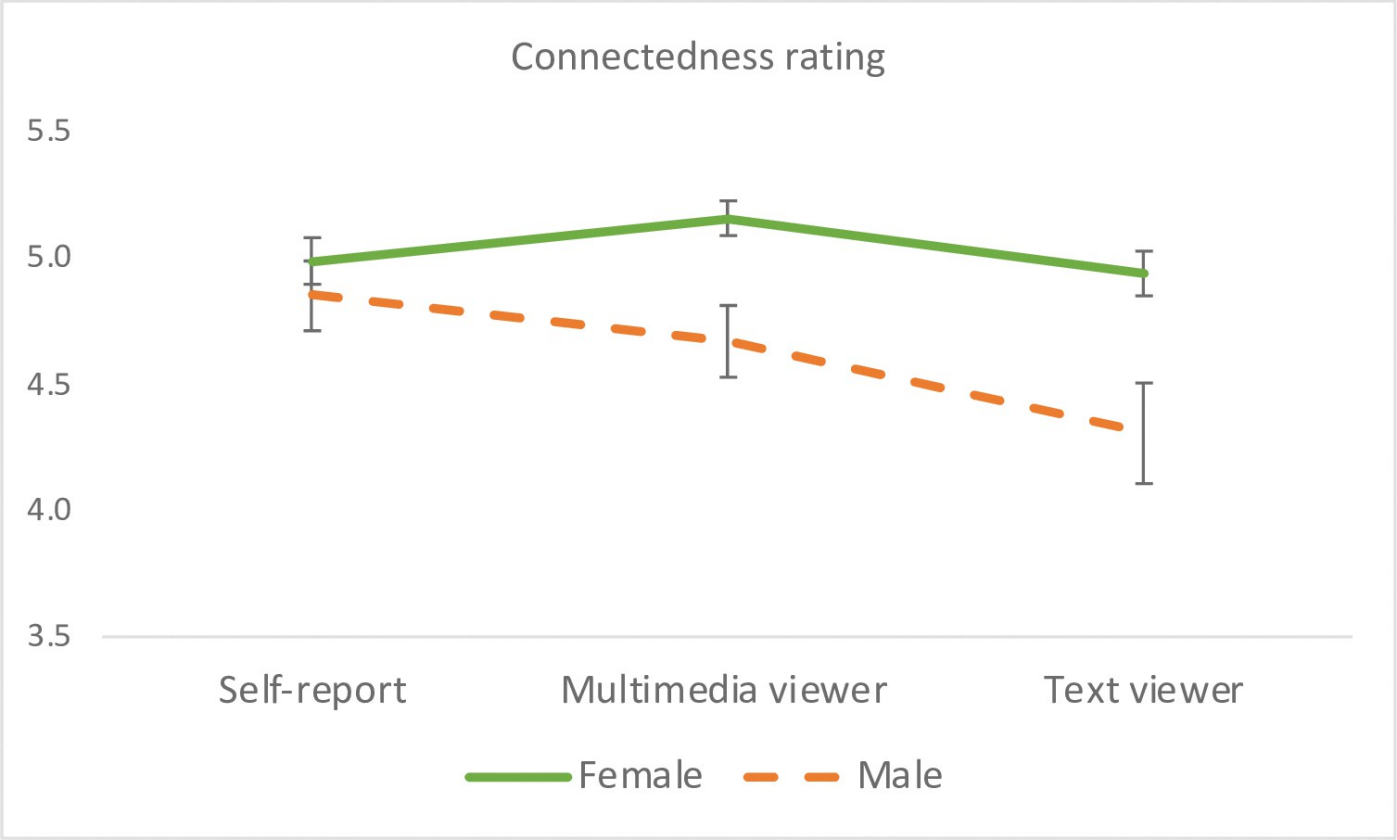
Gender effect on the rating of connectedness. Error bars represent the standard errors of the means. Both viewer groups and participants themselves rated women Facebook users higher than men on connectedness.

A significant rater effect emerged, *F*(2, 149) = 6.20, *p* = .0026, η_p_^2^ = .077, which was driven by the higher rating of the multimedia group than the text group, *F*(1, 150) = 12.48, *p* = .0005, η_p_^2^ = .077. Participants’ self-rating did not differ significantly from either viewer group ratings. When gender was entered into the model, a significant main effect of gender emerged, *F*(1, 149) = 13.59, *p* = .0003, η_p_^2^ = .084, whereby viewers and participants themselves rated women higher than men on connectedness. There was no significant Gender x Rater interaction (see gender effect in Fig 5). Ethnicity had no significant main effect or interaction on connectedness ratings.

#### Self-esteem

**Fig 6:**
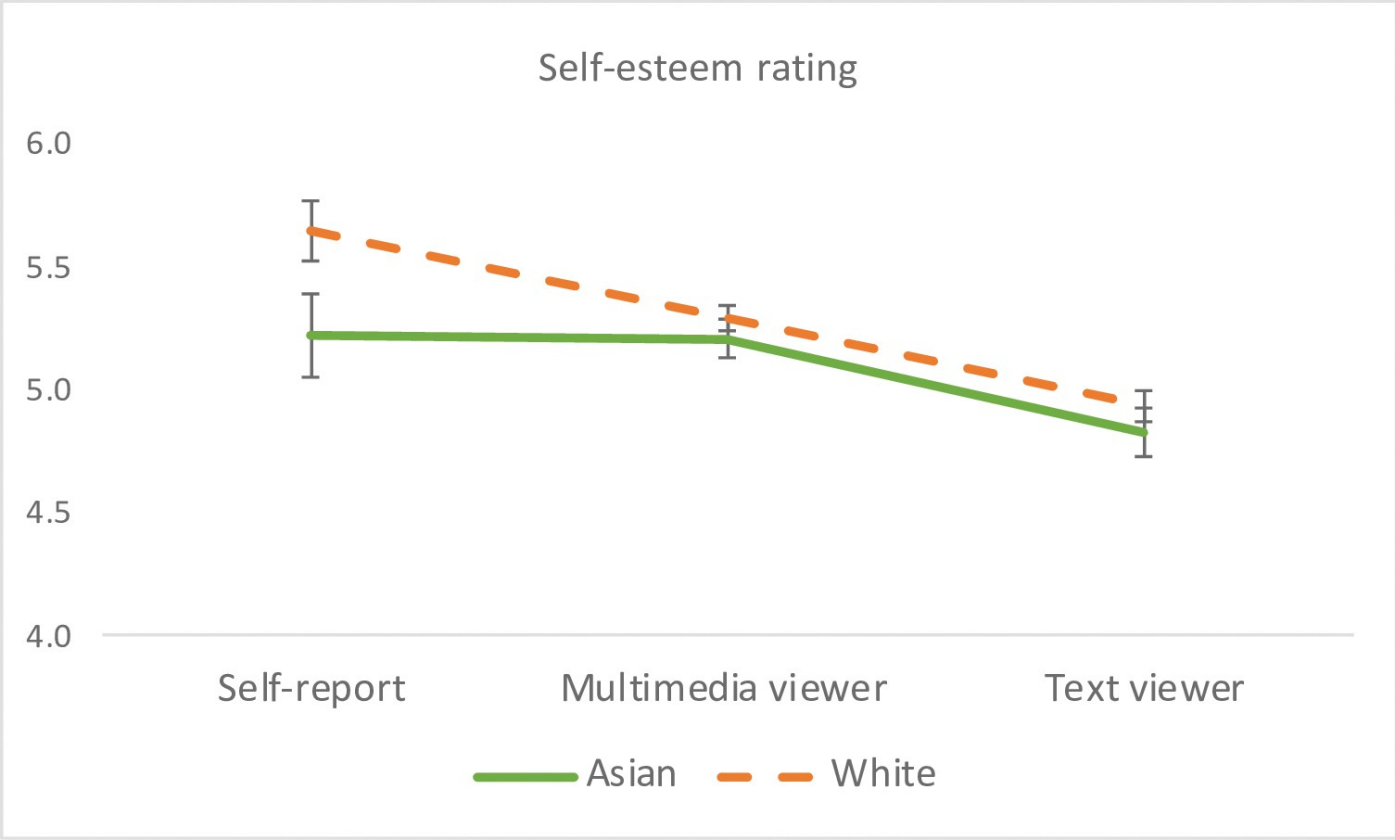
Ethnicity effect on the rating of self-esteem. Error bars represent the standard errors of the means. Both viewer groups and participants themselves rated White Facebook users higher on self-esteem than Asians.

A significant rater effect also emerged for self-esteem, *F*(2, 146) = 36.06, *p* < .0001, η_p_^2^ = .33, whereby participants rated higher on their self-esteem than did the multimedia group, *F*(1, 147) = 7.89, *p* = .0056, η_p_^2^ = .051, and the text group, *F*(1, 148) = 44.63, *p* < .0001, η_p_^2^ = .23. The multimedia group had higher ratings than did the text group, *F*(1, 150) = 50.39, *p* < .0001, η_p_^2^ = .25. Gender had no significant main effect or interaction on self-esteem ratings. When ethnicity was entered into the model, a significant main effect of ethnicity emerged, *F*(1, 107) = 5.52, *p* = .021, η_p_^2^ = .049, whereby viewers and participants themselves rated White participants higher than Asians on self-esteem (see ethnicity effect in Fig 6). The Ethnicity x Rater interaction did not approach significance.

#### Independence

**Fig 7:**
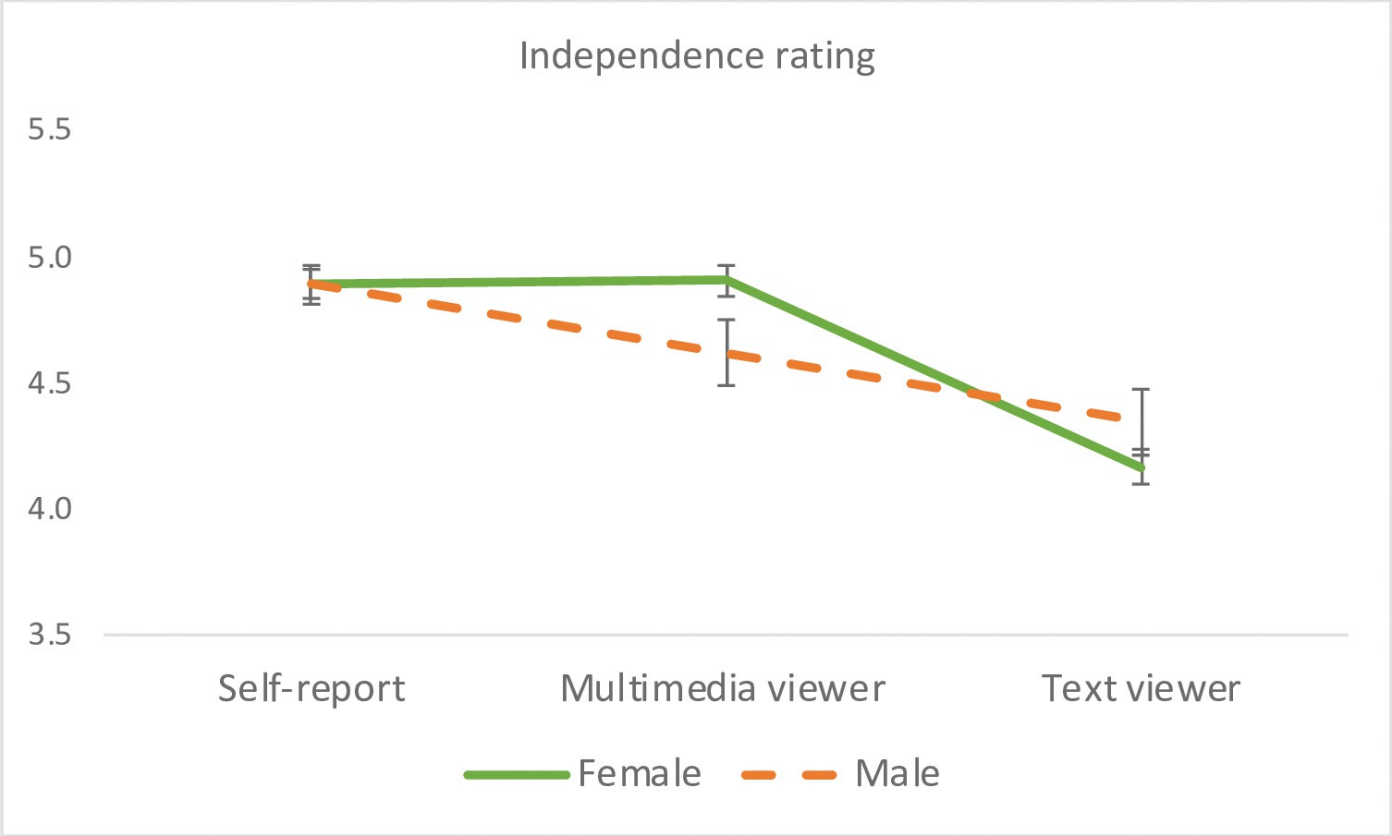
Interaction effect of rater group by gender on independence. Error bars represent the standard errors of the means. Multimedia viewers, but not text viewers or participants themselves, rated women Facebook users higher than men on independence.

A significant rater effect emerged, *F*(2, 149) = 56.03, *p* < .0001, η_p_^2^ = .43, which was driven by the lower rating of the text group than the participants’ self-report, *F*(1, 151) = 71.48, *p* < .0001, η_p_^2^ = .32, and the multimedia group, *F*(1, 150) = 91.14, *p* < .0001, η_p_^2^ = .38, who did not differ significantly from each other. When gender was entered into the model, a significant Gender x Rater interaction emerged, *F*(2, 148) = 5.85, *p* = .0036, η_p_^2^ = .073. Whereas the multimedia group rated women higher than men on independence (*p* < .05), there was no significant gender difference in the text group or participants’ self-report (See Gender x Rater interaction in Fig 7). A significant Ethnicity x Rater interaction also emerged, *F*(2, 109) = 3.37, *p* = .038, η_p_^2^ = .058, although follow-up comparisons showed no significant ethnicity effect within any rating group (*ps* > .05).

#### Interdependence

**Fig 8:**
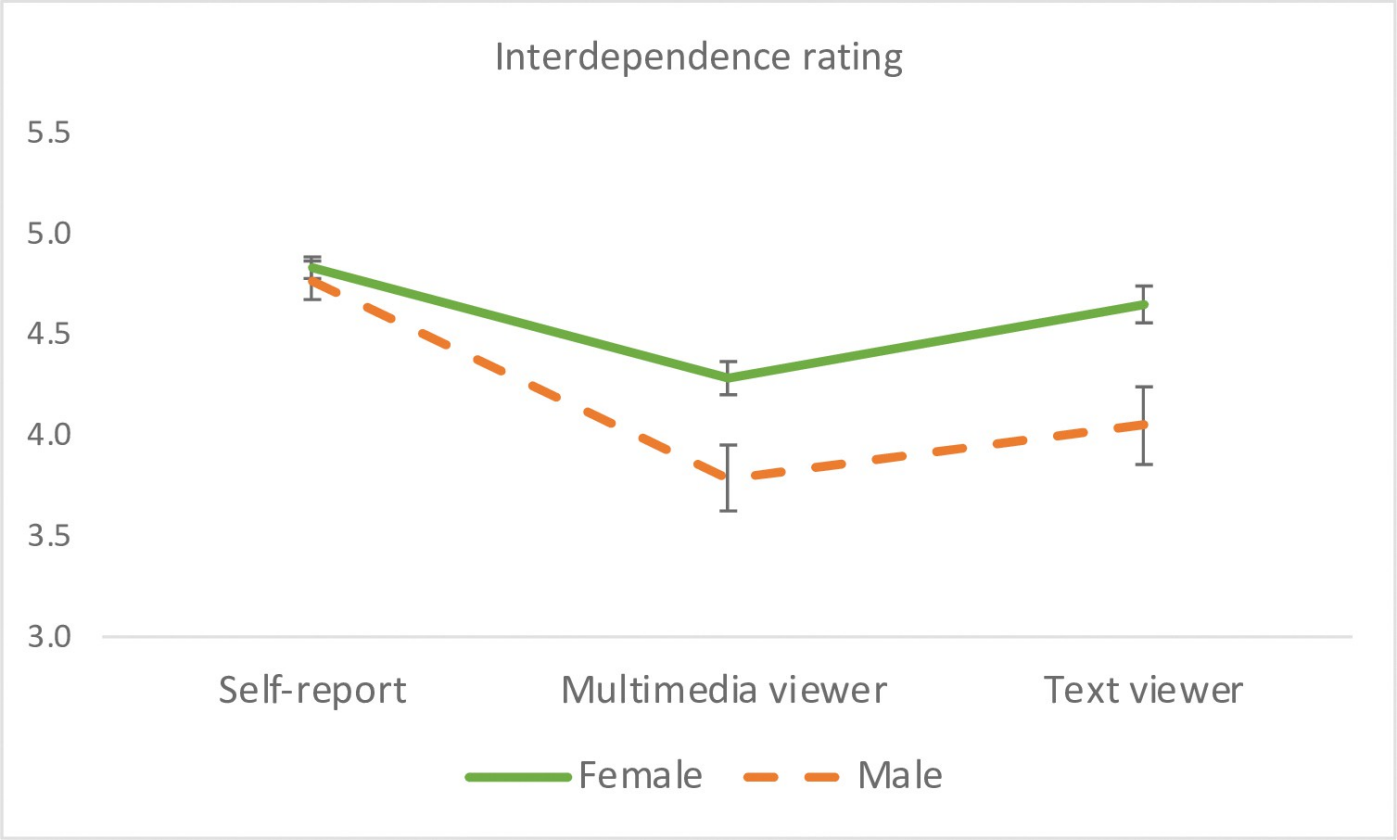
Interaction effect of rater group by gender on interdependence. Error bars represent the standard errors of the means. Both viewer groups, but not participants themselves, rated women Facebook users higher than men on interdependence.

A significant rater effect emerged, *F*(2, 149) = 29.33, *p* < .0001, η_p_^2^ = .28, whereby participants rated higher their interdependence than did the multimedia group, *F*(1, 150) = 56.98, *p* < .0001, η_p_^2^ = .28, and the text group, *F*(1, 151) = 11.72, *p* = .0008, η_p_^2^ = .072. The multimedia group had lower ratings than the text group, *F*(1, 150) = 17.61, *p* < .0001, η_p_^2^ = .11. When gender was entered into the model, a significant main effect of gender, *F*(1, 149) = 12.92, *p* = .0004, η_p_^2^ = .080, and a Gender x Rater interaction emerged, *F*(2, 148) = 3.26, *p* = .041, η_p_^2^ = .042. Both viewer groups rated women higher on interdependence than men (*p*s < .01), who did not significantly differ in their self-reported interdependence (See Gender x Rater interaction in Fig 8). Ethnicity had no significant main effect or interaction on interdependence ratings.

### Facebook users’ self-descriptions and cyber audience’s descriptions of the Facebook users

In this section, we examined the judges’ evaluation of consistency between participants’ (Facebook users’) self-descriptions and the descriptions provided by the two groups of 6 viewers (cyber audience) about the participants based on their Facebook status updates. The mean consistency scores and standard deviations are shown in Table 2 by viewer group.

**Table 2:** Means and standard deviations of consistency scores.

	Multimedia Viewer		Text Viewer	
	Mean	SD	Mean	SD
Consistent	1.85^a^	0.89	1.45^b^	0.84
Inconsistent	0.09^a^	0.13	0.19^b^	0.27
Commission	8.41^a^	0.93	8.61^b^	0.81
Omission	8.42^a^	0.80	8.60^b^	0.88

*Note*: Significant differences in each consistency score are

denoted by different superscripts.

A repeated-measures ANOVA was conducted on each consistency score. The multimedia group provided user-descriptions that were more consistent with the participants’ self-descriptions than did the text group, *F*(1, 149) = 49.60, *p* < .0001, η_p_^2^ = .25. In contrast, the text group provided more inconsistent user-descriptions from the participants’ self-descriptions than did the multimedia group, *F*(1, 149) = 19.56, *p* < .0001, η_p_^2^ = .12. Furthermore, the text group provided more commissions, *F*(1, 149) = 11.60, *p* = .0008, η_p_^2^ = .072, and more omissions, *F*(1, 149) = 11.27, *p* = .0010, η_p_^2^ = .070, in their user-descriptions than did the multimedia group. Thus, compared with viewers who saw the text version of Facebook status updates, viewers who saw the multimedia version formed impressions about the Facebook users more in line with the users’ self-views. Additional analyses with gender and ethnicity included in the models yielded identical patterns of results, with no significant main effect or interaction involving gender or ethnicity.

### Consensus among cyber audience members on Facebook users’ persona

To examine the consensus *within* each viewer group (i.e., cyber audience) in how individual viewers perceived the participants (i.e., Facebook users), Cronbach’s alpha coefficients were calculated within each viewer group for viewer ratings on participants’ characteristics. The coefficients are presented in Table 3.

**Table 3:** Consensus among viewers.

User Characteristics	Multimedia Viewer	Text Viewer
Extroversion	0.76	0.72
Disclosiveness	0.68^a^	0.78^b^
Connectedness	0.75^a^	0.85^b^
Self-esteem	0.49	0.55
Independence	0.46^a^*	0.61^b^*
Interdependence	0.71^a^	0.82^b^

*Note*: Significant differences are denoted by different superscripts.

* Represents marginal significance.

Overall, viewers of both groups showed a moderately high consensus on extroversion, disclosiveness, connectedness, and interdependence (.68 - .85), and a lower consensus on self-esteem and independence (.46 - .61).

The Cronbach’s alpha coefficients were compared between the two viewer groups using the *cocron* package for the R programming language [56]. As shown in Table 3, viewers of the text group were more consistent among themselves than those of the multimedia group in ratings on disclosiveness, *χ*^2^(1) = 6.56, *p* = 0.010, connectedness, *χ*^2^(1) = 10.13, *p* = 0.0015, and interdependence, *χ*^2^(1) = 8.091, *p* = 0.0044. Viewers of the text group were also more consistent in ratings on independence than viewers of the multimedia group at marginal significance, *χ*^2^(1) = 3.23, *p* = 0.072. Thus, viewers of the text group exhibited a generally greater consensus in their perceptions of the Facebook users’ persona than viewers of the multimedia group.

## Discussion

The present study is the first that we know of to examine how social media users’ self-presentations in online posts are perceived by the cyber audience, and how this outsourced meaning-making process is influenced by the format (text versus multimedia) of social media posts and moderated by the gender and ethnicity of the social media users. Taking a cognitive meaning-making perspective, the study yielded important findings that shed new light on the dynamic interaction between social media users and the cyber audience in the construction of a digitally extended self.

As expected, there were substantial discrepancies between how the cyber audience perceived the characteristics of Facebook users based on their status updates and the users’ self-reported personal characteristics (H1). Correlations between self-reports and viewer ratings were overall small, suggesting that there is little consensus between social media users and the cyber audience across individual ratings. Furthermore, the difference between viewer ratings and participants’ self-ratings at the group level was significant across almost all measures. Thus, both individual-level correlations and group-level comparisons suggest little consistency between self-ratings and viewer-ratings of Facebook users’ personal characteristics. These findings contrast with previous research showing that observers are highly accurate in perceiving the personality of social media users based on personal blogs and profiles where personal characteristics are often explicitly and coherently articulated [10, 28, 29]. When processing the seemingly unconnected and disparate social media posts like Facebook status updates, the perception of personal characteristics necessitates an outsourced constructive process of meaning-making in the cyber audience to work out who the author is [1, 6, 27]. This can in turn contribute to discrepancies between the cyber audience’s perceptions of social media users and the users’ self-views. The current findings thus provide an important extension to the literature on person perception online.

Furthermore, the discrepancies between the ratings by the cyber audience and Facebook users varied by specific characteristics (H2): Both viewer groups perceived the participants as more disclosive than did the participants themselves. This is consistent with prior studies showing that the cyber audience is sensitive and responsive to self-disclosure in social media posts [20, 33, 34]. On the other hand, both viewer groups perceived the participants as of lower self-esteem and less interdependent than how the participants viewed themselves, and viewers of the text group also underestimated the participants’ extroversion and independence. The findings suggest that these characteristics may be difficult to infer based on the content of the status updates. Interestingly, the perceptions of connectedness were consistent between the Facebook users and the two viewer groups, a characteristic closely related to the social-relational motive of sharing information online [4, 5, 7]. The cyber audience’s accurate perception of the Facebook users’ connectedness may reflect the notion that posting on social media serves primarily the purpose of connecting with others.

Importantly, the discrepancies between viewer ratings and participants’ self-ratings were significant for 3 out of the 6 measures for the multimedia group, and 5 out of the 6 measures for the text group. These findings suggest that viewers of the multimedia group generated relatively more accurate information about the Facebook users than viewers of the text group (H3). This is further confirmed by the consistency scores between participants’ self-descriptions and the descriptions provided by the viewers about the participants. Compared with the text group viewers, the multimedia group viewers provided more consistent descriptions and fewer inconsistent descriptions, fewer commissions, and fewer omissions. These findings suggest that the multimedia group formed impressions about the Facebook users’ persona more in line with the users’ self-views than did the text group. Compared with text-only posts, multimedia posts allow social media users to communicate themselves via diverse means such as photos, videos, and hyperlinks in addition to text, thus containing richer information about who the author is [6, 19, 36, 37]. This appears to facilitate the cyber audience’s meaning making of the online posts to form accurate impressions about the social media users.

However, viewers of the text group seemed more sensitive to individual variations, whereby their ratings were significantly correlated with the participants’ self-reports for 3 out of the 6 measures, relative to 1 measure by the multimedia group. Furthermore, viewers of the text group exhibited a greater consensus in their ratings on 4 out of the 6 characteristics of the Facebook users than did viewers of the multimedia group (H4). It is possible that the diverse forms of information in multimedia posts may result in more individual differences in the meaning-making process and, in turn, contribute to more inconsistent views among the cyber audience members, when compared with text-only posts. Notably, there was an overall good consensus within each viewer group across most measures, consistent with prior findings of personality perception based on personal blogs and profiles [10, 28, 29]. It is further in line with the notion of the transactive mind of the virtual community, where the cyber audience members engage in a shared process of meaning-making to derive information from diverse social media posts about who the author is [6, 27, 31, 32]. In the real-life social media context, the cyber audience members often interact with each other and exchange information about the author, which can further contribute to shared views in the virtual community about the author’s persona [1].

Some interesting results emerged pertaining to gender and ethnicity (H5), in line with findings of personality judgments in offline contexts [39]. Both viewer groups perceived greater extraversion in female than male Facebook users, which is in line with the general finding that women tend to score higher on extraversion than men [42]. Also consistent with the gendered self-construal [38, 40, 41], viewers perceived greater connectedness and interdependence in female than male Facebook users. Viewers of the multimedia group also rated women higher than men on independence. Interestingly, Facebook users themselves did not show gender differences in their self-reports in any of these personal characteristics except connectedness. Given that the viewers were blind to the participants’ names and other identifiable information, these findings may suggest that social media users present themselves in online posts generally in line with their gendered characteristics and that women may be more expressive than men in their online posts. The findings further suggest that the cyber audience can derive these gendered characteristics through meaning making of the social media users’ posts, especially with the assistance of multimedia tools.

Similarly, in line with prior findings that Westerners such as White Americans tend to score higher on extraversion than Asians [43, 44], viewers of the multimedia group perceived White Facebook users as more extroverted than Asians, although Facebook users did not differ in their self-reports. Both groups of viewers and Facebook users themselves perceived White Facebook users as of higher self-esteem than Asians. This is consistent with the notion that Western cultures place a greater value on self-enhancement and positive self-regard than East Asian cultures, and that individuals in turn view and present themselves within their respective cultural frameworks [45–47]. Taken together, the findings suggest the impact of culture on the dynamic interaction between social media users’ private self-views, their self-presentations online, and the cyber audience’s meaning making of the users’ social media posts.

Although the current studies yielded original findings on how Facebook users’ persona was perceived by the cyber audience based on status updates and the factors that influence the audience’s meaning-making process, there are important limitations. In particular, the viewers only read the 20 most recent status updates of the Facebook users. It is possible that they can form more accurate perceptions of the Facebook users’ persona based on a longer history of status updates. Also, the viewers did not see comments on the status updates or share information with each other as they might in actual online contexts. Future research that considers these factors will reveal additional nuances of the cyber audience’s meaning making of online posts. In addition, the current study focused on Facebook users’ personal and demographic characteristics in relation to the cyber audience’s perception of their persona based on status updates. Future research should examine the characteristics of the cyber audience in influencing how social media users are perceived, and also study the outsourced meaning-making process on other popular social media sites such as Instagram and TikTok. Additional research should also examine the contribution of different media formats to impression formation as new technological features continue being introduced to social media platforms for individuals to share their experiences online.

In conclusion, the current study yielded original findings on the dynamic interplay between social media users and the cyber audience in impression formation based on online posts. Although authentic self-presentation online is associated with many psychological benefits, it may not be accurately perceived by others in the process of making sense of the social media users’ posts, especially for certain personal characteristics not easily inferable from the posts. The presentational formats further differentially influence the accuracy and consensus of the cyber audience’s meaning making of the users’ persona. Still, cyber audience members are better at inferring user characteristics that serve social motives, and they show an overall good consensus in their person perceptions. They also appear to be able to derive some of the gendered and cultured characteristics of the Facebook users from the status updates. Taken together, with the meaning-making process outsourced to the cyber audience, the construction of a digitally extended self of the social media users is a dynamic process that takes place not only through user self-presentations but also in the transactive mind of the virtual community.

## References

[1] Wang Q. (2022). The triangular self in the social media era. *Memory*, *Mind & Media*, 1, E4, 1–12. 10.1017/mem.2021.6

[2] Bazarova N. N., Choi Y. H., Whitlock J., Cosley D., & Sosik V. (2017). Psychological distress and emotional expression on Facebook. *Cyberpsychology*, *Behavior*, *and Social Networking*, 20 *(*3*)*, 157–163. psyh. doi: 10.1089/cyber.2016.0335 28117594

[3] Goffman E. (1956). *The Presentation of Self in Everyday Life*. New York, NY: Doubleday.

[4] Hollenbaugh E. E. (2011). Motives for maintaining personal journal blogs. *CyberPsychology*, *Behavior & Social Networking*, 14 *(*1/2*)*, 13–20. doi: 10.1089/cyber.2009.0403 21329438

[5] Stone C. B., Guan L., LaBarbera G., Ceren M., Garcia B., Huie K., et al. (2022). Why do people share memories online? An examination of the motives and characteristics of social media users. *Memory*. doi: 10.1080/09658211.2022.2040534 35193451

[6] Wang Q. (2013a). *The autobiographical self in time and culture*. New York, NY: Oxford University Press. doi: 10.1093/acprof:oso/9780199737833.001.0001

[7] Wang Q. (2020). Creation of the Purposes of Online Memory Sharing Scale. *International Journal of Applied Psychology*, 10 *(*3*)*. 10.31234/osf.io/jh5sx

[8] Bargh J. A., McKenna K. Y. A., & Fitzsimons G. M. (2002). Can you see the real me? Activation and expression of the “true self” on the Internet. *Journal of Social Issues*, 58, 33–48.

[9] Markey P. M., & Wells S. M. (2002). Interpersonal perception in Internet chat rooms. *Journal of Research in Personality*, 36, 134–146.

[10] Vazire S., & Gosling S. D. (2004). e-Perceptions: Personality Impressions Based on Personal Websites. *Journal of Personality and Social Psychology*, 87(1), 123–132. doi: 10.1037/0022-3514.87.1.123 15250797

[11] Bazarova N. N., Taft J. G., Choi Y. H., & Cosley D. (2013). Managing impressions and relationships on Facebook: Self-presentational and relational concerns revealed through the analysis of language style. *Journal of Language and Social Psychology*, 32 *(*2*)*, 121–141. 10.1177/0261927X12456384

[12] Stefanone M. A., & Jang C-Y (2008). Writing for Friends and Family: The Interpersonal Nature of Blogs. *Journal of Computer-Mediated Communication*, 13(1), 123–140.

[13] Miura A., & Yamashita K. (2007). Psychological and social influences on blog writing: An online survey of blog authors in Japan. *Journal Of Computer-Mediated Communication*, 12 *(*4*)*, 1452–1471.

[14] Sheldon K. M., Abad N., & Hinsch C. (2011). A two-process view of Facebook use and relatedness need-satisfaction: Disconnection drives use, and connection rewards it. *Journal of Personality and Social Psychology*, 100, 4, 766–775 doi: 10.1037/a0022407 21280967

[15] Faelens L., Hoorelbeke K., Fried E., De Raedt R., & Koster E. H.W. (2019). Negative influences of Facebook use through the lens of network analysis. *Computers in Human Behavior*, 96, 13–22. 10.1016/j.chb.2019.02.002.

[16] Gonzales A. L., & Hancock J. T. (2011). Mirror, mirror on my Facebook wall: Effects of exposure to Facebook on self-esteem. *Cyberpsychology*, *Behavior*, *and Social Networking*, 14(1–2), 79–83. doi: 10.1089/cyber.2009.0411 21329447

[17] Wang Q., Blenis R. C., Ng M., & Gonzalez P. (2015). Going public: The impact of social media on autobiographical memory. *Poster session presented at the 27th APS Annual Convention*, *New York*, *NY*.

[18] Oh H. J., & LaRose R. (2016). Impression management concerns and support-seeking behavior on social network sites. *Computers in Human Behavior*, 57, 38–47. 10.1016/j.chb.2015.12.005

[19] Serfaty V. (2004). *The mirror and the veil*: *An overview of American online diaries and blogs*. New York: Rodopi.

[20] Chen H-T, & Li X. (2017). The contribution of mobile social media to social capital and psychological well-being: Examining the role of communicative use, friending and self-disclosure. Computers in Human Behavior, 75, 958–965. doi: 10.1016/j.chb.2017.06.011

[21] Ko H-C, & Pu H-J(2010). Can blogging enhance resilience through self-disclsoure? A positive net perspective. *Review of Business Research*, 10, 2, 28–36.

[22] Forest A. L., & Wood J. V. (2012). When social networking is not working: Individuals with low self-esteem recognize but do not reap the benefits of self-disclosure on Facebook. *Psychological Science*, 23, 295–302. doi: 10.1177/0956797611429709 22318997

[23] Hou Y., Xiong D., Jiang T., Song L., & Wang Q. (2019). Social media addiction: Its impact, mediation, and intervention. *Cyberpsychology*, 13 *(*1*)*, 4. doi: 10.5817/CP2019-1-4

[24] Habermas T., & Bluck S. (2000). Getting a life: The emergence of the life story in adolescence. *Psychological Bulletin*, 126, 748–769. doi: 10.1037/0033-2909.126.5.748 10989622

[25] McAdams D. P. (2006). *The redemptive self*: *Stories Americans live by*. New York: Oxford University Press.

[26] Singer J. A. (2004). Narrative identity and meaning making across the adult lifespan: An introduction. *Journal of Personality*, 72, 437–460. doi: 10.1111/j.0022-3506.2004.00268.x 15102034

[27] Page R. (2010). Re-examining narrativity: Small stories in status updates. *Text & Talk*, 30–4, 423–444.

[28] Back M. D., Stopfer J. M., Vazire S., Gaddis S., Schmukle S. C., Egloff B., et al. (2010). Facebook profiles reflect actual personality, not self-idealization. *Psychological Science*, 21, 372–374. doi: 10.1177/0956797609360756 20424071

[29] Tskhay K. O., & Rule N. O. (2014). Perceptions of personality in text-based media and OSN: A meta-analysis. Journal of Research in Personality, 49, 25–30. 10.1016/j.jrp.2013.12.004

[30] Mickes L, Darby RS, Hwe V, Bajic D, Warker JA, Harris CR, et al. (2013). Major memory for microblogs. *Memory & Cognition*, 41(4), 481–9. doi: 10.3758/s13421-012-0281-6 23315488

[31] Wegner D. M. (1986). Transactive memory: A contemporary analysis of the group mind. In Mullen B. & Goethals G. R.(Eds.), *Theories of group behavior* *(pp*. 185–208). NewYork, NY: Springer-Verlag.

[32] Wegner D. M. (2012). Don’t Fear the Cybermind. *New York Times*. https://www.nytimes.com/2012/08/05/opinion/sunday/memory-and-the-cybermind.html

[33] Hong S., Jahng M.R., Lee N., & Wise K. (2020). Do you filter who you are?: Excessive self-presentation, social cues, and user evaluations of Instagram selfies. *Computer in Human Behavior*, 104, 106159. 10.1016/j.chb.2019.106159

[34] Walsh R.M., Forest A.L., & Orehek E. (2020). Self-disclosure on social media: The role of perceived network responsiveness. *Computer in Human Behavior*, 104, 106162. 10.1016/j.chb.2019.106162

[35] Stsiampkouskaya K., Joinson A., Piwek L., & Ahlbom C. (2021) Emotional responses to likes and comments regulate posting frequency and content change behaviour on social media: An experimental study and mediation model, *Computers in Human Behavior*, 124, 106940. 10.1016/j.chb.2021.106940.

[36] Hall J. A, Pennington N., & Lueders A. (2013). Impression management and formation on Facebook: A lens model approach. New Media and Society. 10.1177/1461444813495166.

[37] Ivcevic Z., & Ambady N. (2012). Personality impressions from identity claims on Facebook. *Psychology of Popular Media Culture*, 1(1), 38–45. 10.1037/a0027329

[38] Cross S.E., & Madson L. (1997). Models of the self: Self-construals and gender. *Psychological Bulletin*. 122 *(*1*)*, 5–37. doi: 10.1037/0033-2909.122.1.5 9204777

[39] Gosling S. D., Ko S. J., Mannarelli T., & Morris M. E. (2002). A room with a cue: Personality judgments based on offices and bedrooms. *Journal of Personality and Social Psychology*, 82(3), 379–398. doi: 10.1037//0022-3514.82.3.379 11902623

[40] Wang Q. (2013b). Gender and emotion in everyday event memory. *Memory*, 21, 503–511. doi: 10.1080/09658211.2012.743568 23190136

[41] Zaman W., & Fivush R. (2011). When my mom was a little girl.: gender differences in adolescents’ intergenerational and personal stories. *Journal of Research on Adolescence*, 21, 703–716. 10.1111/j.1532-7795.2010.00709.x.

[42] Weisberg Y.J., DeYoung C.G., & Hirsh J.B. (2011). Gender differences in personality across the ten aspects of the Big Five. *Frontiers in Psychology*, 2,178. doi: 10.3389/fpsyg.2011.00178 21866227 PMC3149680

[43] Oyserman D., Coon H. M., & Kemmelmeier M. (2002). Rethinking individualism and collectivism: Evaluation of theoretical assumptions and meta-analyses. *Psychological Bulletin*, 128, 3–72. doi: 10.1037/0033-2909.128.1.3 11843547

[44] Song H., & Kwon N. (2012). The relationship between personality traits and information competency in Korean and American students. *Social Behavior and Personality*, 40, 1153–1162. doi: 10.2224/sbp.2012.40.7.115

[45] Hamamura T. (2017) Cultural differences in self-esteem. In Zeigler-Hill V., Shackelford T. (eds), *Encyclopedia of Personality and Individual Differences*. Springer, Cham. 10.1007/978-3-319-28099-8_1126-1

[46] Heine S. J., & Hamamura T. (2007). In search of east Asian self-enhancement. *Personality and Social Psychology Review*, 11(2), 204. doi: 10.1177/1088868306294587 18453453

[47] Jeon H. J., Wang Q., Burrow A. L., & Ratner K (2020). Perspectives of future health in self and others: The moderating role of culture. *Journal of Health Psychology*, 25 *(*5*)*, 703–712. doi: 10.1177/1359105317730897 28929826

[48] Faul F., Erdfelder E., Buchner A., & Lang A.-G. (2009). Statistical power analyses using G*Power 3.1: Tests for correlation and regression analyses. *Behavior Research Methods*, 41, 1149–1160. doi: 10.3758/BRM.41.4.1149 19897823

[49] Kuhn M. H., & McPartland T. S. (1954). An empirical investigation of self-attitudes. *American Sociological Review*, 19 *(*1), 68–76. 10.2307/2088175

[50] Wang Q. (2001). Cultural effects on adults’ earliest childhood recollection and self-description: Implications for the relation between memory and the self. *Journal of Personality and Social Psychology*, 81, 220–233. 11519928 10.1037//0022-3514.81.2.220

[51] McCrae R. R., & Costa P. T. Jr. (2008). *The five-factor theory of personality*. In John O. P., Robins R. W, & Pervin A.(Eds.), *Handbook of Personality*: *Theory and Research* (p. 159–181). The Guilford Press.

[52] Wheeless L.R. (1978). A follow-up study of the relationships among trust, disclosure, and interpersonal solidarity. *Human Communication Research*, 4, 143–57.

[53] Rosenberg M. (1965). *Society and the adolescent self-image*. Princeton, NJ: Princeton University Press.

[54] Singelis T. M. (1994). The measurement of independent and interdependent self-construals. *Personality and Social Psychology Bulletin*, 20, 580–591.

[55] Talarico J. M., & Rubin D. C. (2003). Confidence, not consistency, characterizes flashbulb memories. *Psychological Science*, 14, 455–461. doi: 10.1111/1467-9280.02453 12930476

[56] Diedenhofen B., & Musch J. (2016). cocron: A web interface and R package for the statistical comparison of Cronbach’s alpha coefficients. International Journal of Internet Science, 11, 51–60. http://comparingcronbachalphas.org.

